# Whole Genome Sequencing Results Associated with Minimum Inhibitory Concentrations of 14 Anti-Tuberculosis Drugs among Rifampicin-Resistant Isolates of *Mycobacterium Tuberculosis* from Iran

**DOI:** 10.3390/jcm9020465

**Published:** 2020-02-07

**Authors:** Jalil Kardan-Yamchi, Hossein Kazemian, Simone Battaglia, Hamidreza Abtahi, Abbas Rahimi Foroushani, Gholamreza Hamzelou, Daniela Maria Cirillo, Arash Ghodousi, Mohammad Mehdi Feizabadi

**Affiliations:** 1 Division of Microbiology, Department of Pathobiology, School of Public Health, Tehran University of Medical Sciences, Tehran 1417653911, Iran; jalil_kardan@outlook.com; 2 Department of Microbiology, School of Medicine, Tehran University of Medical Sciences, Tehran 1417653911, Iran; h.kazemian@outlook.com; 3 Emerging Bacterial Pathogens Unit, Division of Immunology, Transplantation and Infectious Diseases, IRCCS San Raffaele Scientific Institute, 20132 Milan, Italy; battaglia.simone@hsr.it (S.B.);; 4 Thoracic Research Center, Imam Khomeini Hospital Complex, Tehran University of Medical Sciences, Tehran 1417653911, Iran; hrabtahi@gmail.com; 5 Department of Epidemiology and Biostatistics, School of Public Health, Tehran University of Medical Sciences, Tehran 1417653911, Iran; rahimifo@tums.ac.ir; 6 Tehran Regional Reference Laboratory for Tuberculosis, Tehran University of Medical Sciences, Tehran 1417653911, Iran; hamzehloo2@yahoo.com

**Keywords:** *Mycobacterium tuberculosis*, drug resistance, mutation, gene, minimum inhibitory concentration, whole genome sequencing

## Abstract

Accurate and timely detection of drug resistance can minimize the risk of further resistance development and lead to effective treatment. The aim of this study was to determine the resistance to first/second-line anti-tuberculosis drugs in rifampicin/multidrug-resistant *Mycobacterium tuberculosis* (RR/MDR-MTB) isolates. Molecular epidemiology of strains was determined using whole genome sequencing (WGS)-based genotyping. A total of 35 RR/MDR-MTB isolates were subjected to drug susceptibility testing against first/second-line drugs using 7H9 Middlebrook in broth microdilution method. Illumina technology was used for paired-end WGS applying a Maxwell 16 Cell DNA Purification kit and the NextSeq platform. Data analysis and single nucleotide polymorphism calling were performed using MTBseq pipeline. The genome-based resistance to each drug among the resistant phenotypes was as follows: rifampicin (97.1%), isoniazid (96.6%), ethambutol (100%), levofloxacin (83.3%), moxifloxacin (83.3%), amikacin (100%), kanamycin (100%), capreomycin (100%), prothionamide (100%), D-cycloserine (11.1%), clofazimine (20%), bedaquiline (0.0%), and delamanid (44.4%). There was no linezolid-resistant phenotype, and a bedaquiline-resistant strain was wild type for related genes. The Beijing, Euro-American, and Delhi-CAS were the most populated lineage/sublineages. Drug resistance-associated mutations were mostly linked to minimum inhibitory concentration results. However, the role of well-known drug-resistant genes for D-cycloserine, clofazimine, bedaquiline, and delamanid was found to be more controversial.

## 1. Introduction

Multidrug-resistant tuberculosis (MDR-TB), with a wide range of prevalence around the world is considered as a global public health concern [1]. In 2017, approximately 558,000 (483,000–639,000) rifampicin-resistant tuberculosis (RR-TB) cases were estimated, of which 82% were MDR. Overall, 3.5% of new cases and 18% of previously treated cases were RR/MDR-TB and reported to be 1.3% and 8.3%, respectively, in Iran [2]. However, drug resistance in the neighboring countries of Iran is remarkably higher, as in Afghanistan, Armenia, and Azerbaijan, MDR incidence was 4.1%, 11%, and 13% among new cases, and it was 16%, 47%, and 39% among previously treated patients, respectively [2].

Treatment of patients with MDR-TB is more time-consuming and requires expensive drugs with more toxicity, compared to drug-susceptible TB. Additionally, inappropriate and inadequate treatment increases the risk of resistance to other drugs, possible transmission, and prolonged treatment [3]. In other words, accurate drug susceptibility testing (DST) is required to achieve effective treatment and minimize the risk of further resistance development [4].

Spontaneous mutations in the genes encoding drug targets or drug-converting enzymes are the main cause of acquired resistance in *Mycobacterium tuberculosis* complex (MTBC) [3]. Other mechanisms can also be involved, such as drug efflux pumps [5]. Several phenotypic DST (pDST) methods have become available and are done on bacterial cultures after a positive primary culture of the specimen, and thus detecting resistance is technically complex and time-consuming [6]. Furthermore, these techniques have their own problems with test standardization and stability of various antibiotics in different media [7]. Detection of mutations in drug-resistant conferring genes has been proposed as a fast tool for identifying resistance against anti-tuberculosis drugs. However, the sensitivity of molecular methods to detect the resistance of different drugs is debatable [8]. Molecular techniques like Xpert MTB/RIF (Cepheid, Sunnyvale, CA, USA) and line probe assays did not cover all the drugs (e.g., linezolid, bedaquiline, delamanid, etc.) and have high sensitivities for drugs which only a few mutations within a gene are responsible for resistance (e.g., rifampicin) [9]. Additionally, these methods show low sensitivity for heteroresistant strains with low frequencies of mutant variants [10].

Unfortunately, quantitative phenotypic DST (MICs) data are often limited and genetic background of resistance for some antibiotics were not clearly understood [11]. Accurate identification of chromosomal mutations is accessible using whole genome sequencing (WGS) and linking with the minimum inhibitory concentrations are necessarily needed to better infer the susceptible strains from the resistant ones [12].

This cross sectional study was conducted to determine the minimum inhibitory concentrations (MICs) of 14 anti-tuberculosis drugs, including first-line drugs (rifampicin (RIF), isoniazid (INH), ethambutol (EMB)), fluoroquinolones (levofloxacin (LFX), moxifloxacin (MFX)), second-line injectable drugs (amikacin (AMK), kanamycin (KAN), capreomycin (CAP)), other second-line drugs (prothionamide (PTO), D-cycloserine (DCS), clofazimine (CFZ), linezolid (LZD)), and add-on agents (bedaquiline (BDQ), and delamanid (DLM)). WGS was used to evaluate genotypic resistance of RR/MDR isolates of *Mycobacterium tuberculosis* against aforementioned drugs. Furthermore, sensitivity, specificity, and accuracy of the WGS versus pDST were evaluated to genome-based drug resistance prediction. Diversity of MTB lineages was assigned using WGS-based genotyping to study molecular epidemiology of strains.

## 2. Materials and Methods

### 2.1. Study Design and Mycobacterial Strains

Rifampicin-resistant *M. tuberculosis* strains isolated from patients referred to Tehran Regional Reference Laboratory for Tuberculosis (TRL-Tehran) between 2014 and 2018 were included in the present study. *M. tuberculosis* was identified by biochemical tests after positive primary culture on Lowenstein Jensen (LJ) medium [13]. Resistance to the first-line drugs was determined by the proportion method [14]. In brief, a 1.0 McFarland suspension of bacterial colonies were prepared and dilutions of 10^−2^ and 10^−4^ were inoculated onto LJ medium with 40 µg/mL RIF (Sigma Aldrich, St. Louis, MO, USA) and without drug as test control. After 21 days of incubation at 37 °C, the results were considered sensitive when the proportion of up to 1% of colony growth was seen in the tubes containing the drug. TRL-Tehran undergoes annual external quality control by the World Health Organization (WHO) TB Supranational Reference Laboratory Network in Stockholm, Sweden. Selected isolates were transported to the TB Supranational Reference Laboratory (SRL-Milan) to undergo WGS.

### 2.2. Broth Microdilution

All drugs were purchased from Sigma Aldrich except for BDQ (BOC Sciences, New York, NY, USA) and DLM (Toronto Research Chemicals, North York, ON, Canada). The MICs of first- and second-line drugs were determined by broth micro dilution method using 96-well microplates with OADC (Oleic Albumin Dextrose Catalase) supplemented 7H9 Middlebrook broth [4]. In summary, the final bacterial concentrations were a 10^−2^ dilution of prepared 1.0 MacFarland bacterial suspensions. Medium without any antibiotics inoculated with bacterial suspensions used as growth control wells and negative controls were not inoculated. The plates were sealed in plastic bags and incubated 14 days at 37 °C. Growth and negative-control wells were checked to ensure adequate growth and no contamination, respectively. The first well without any visible growth was as the minimum drug concentration could inhibit *M. tuberculosis* growth. The 2-fold serially diluted drug concentration ranges were as following; 0.12–16 µg/mL for RIF, 0.02–3.2 for INH, 0.25–32 µg/mL for EMB, 0.12–16 µg/mL for LFX, 0.03–4 µg/mL for MFX, 0.06–32 µg/mL for AMK, 0.15–20 µg/mL for KAN, 0.3–40 µg/mL for CAP, 0.3–40 µg/mL for PTO, 0.5–64 µg/mL for DCS, 0.12–16 µg/mL for CFZ, 0.03–8 µg/mL for LZD, 0.015–4 µg/mL for BDQ, and 0.015–3.84 µg/mL for DLM. Since there were no well-defined critical concentrations (CCs) of all anti-TB drugs for drug susceptibility testing on 7H9 broth, drug concentration ranges and the assumed CCs for tested drugs were based on the previous literature [15,16,17]. This experiment was performed in duplicate and *M. tuberculosis* strain H37Rv ATCC 27294 was used for quality control.

### 2.3. Whole Genome Sequencing and Bioinformatic Analysis

At SRL-Milan, libraries were prepared from extracted genomic DNA. All the samples were subcultured in Middlebrook 7H9 broth in order to perform DNA extraction using Maxwell 16 Cell DNA Purification kit (Promega) and put on a single run for 2 × 150 bp run on the Illumina (San Diego, CA, USA) NextSeq 500 next generation sequencing platform as instructed by the manufacturer. All datasets reached a mean genomic coverage depth of at least 50-fold. Sequenced reads were submitted to the NCBI sequence read archive. Data analysis, single nucleotide polymorphism (SNP) calling, and lineage/sublineage classification were performed using MTBseq pipeline [18]. In MTBseq, to facilitate phylogenetic analysis, variant subsets are automatically generated, filtered for repetitive regions [19] and resistance-associated genes, the kind of variant detected, and the presence of other variants within a window of 12 bp within the same dataset [20]. The genome of strain H37Rv (NC-000962.3) was used as a reference. To detect possible resistant subpopulations, the parameters in MTBseq were adjusted to detect variants with low frequency (at least 2-fold coverage and 5% allele frequency). The analysis for genomic DST (gDST) focused on the following genes and their promoter/upstream regions (*rpoB* (DNA-directed RNA polymerase subunit beta), *katG* (catalase-peroxidase), *inhA* (NADH-dependent enoyl-[ACP] reductase), *ahpC* (alkyl hydroperoxide reductase subunit AhpC), *fabG1* (3-oxoacyl-ACP reductase FabG), *furA* (ferric uptake regulation protein FurA), *embA* (arabinosyltransferase A), *embB* (arabinosyltransferase B), *gyrA* (DNA gyrase subunit A), *gyrB* (DNA gyrase subunit B), *rrs* (16S ribosomal RNA), *eis* (enhanced intracellular survival protein), *tlyA* (16S/23S rRNA (cytidine-2′-O)-methyltransferase TlyA), *ethA* (monooxygenase EthA), *ndh* (NADH dehydrogenase), *ald* (L-alanine dehydrogenase), *ddlA* (D-alanine--D-alanine ligase), *alr* (alanine racemase), *cycA* (D-serine/alanine/glycine transporter protein CycA), *Rv0678* (hypothetical protein), *Rv1979c* (permease), *pepQ* (cytoplasmic peptidase PepQ), *rplC* (50S ribosomal protein L3), *rrl* (23S ribosomal RNA), *atpE* (ATP synthase subunit C), *ddn* (deazaflavin-dependent nitroreductase), *fbiA* (probable F420 biosynthesis protein FBIA), *fbiB* (coenzyme F420:L-glutamate ligase), *fbiC* (probable F420 biosynthesis protein FBIA), and *fgd1* (F420-dependent glucose-6-phosphate dehydrogenase)) that have been known to cause resistance to anti-TB drugs, from which the known polymorphisms not correlated with resistance were excluded (i.e., *katG* 2154724 Arg463Leu and *ahpC* promoter 2726105 G-88A) [21]. The genotype for each genome was assigned using Coll nomenclature [22,23,24].

SNP positions, with a reliable base call (10-fold coverage and 75% allele frequency) in at least 95% of the isolates, covered in all samples were concatenated to a sequence alignment, excluding SNPs within a window of 12 bp from each other in the same isolate. From the aligned sequences of concatenated SNPs of all 35 isolates, maximum likelihood trees were calculated using RAxML version 8 [25], with a general time reversible (GTR) substitution model, 1000 resamples, and Gamma20 likelihood optimization to account for rate heterogeneity among sites. The consensus tree was rooted with the “midpoint root” option in FigTree and annotated using the Evolview software. All raw sequencing reads have been deposited at Bioproject at NCBI (project accession nr: PRJNA566379).

### 2.4. Statistical Analysis

Statistical analysis was performed using MedCalc for Windows, version 18.10 (MedCalc Software, Ostend, Belgium), to define the sensitivity, specificity, and accuracy of WGS in predicting each tested drug resistance, compared to the pDST.

## 3. Results

### 3.1. Mycobacterial Strains

In total, 37 RR-MTB strains were collected among the approximately 845 positive cultures for *M. tuberculosis* examined for RIF resistance using proportion method. This includes all the new cases and pretreated patients’ specimens. Based on the MICs results, 35 out of 37 examined strains were confirmed as rifampicin-resistant, and the remaining two isolates, which were susceptible to RIF, were excluded from further analysis. Out of the 35 RR-MTB strains included, one strain was isolated from an extrapulmonary abscess specimen and all others were from pulmonary TB patients.

### 3.2. Minimum Inhibitory Concentrations

MIC (µg/mL) results for H37Rv strain were as following; RIF = 0.12, INH = 0.02, EMB = 1, LFX = 0.25, MFX = 0.03, AMK = 0.25, KAN = 0.6, CAP = 0.6, PTO = 1.25, DCS = 16, CFZ = 0.12, LZD = 0.25, BDQ = 0.015, and DLM = < 0.015.

**Figure 1:**
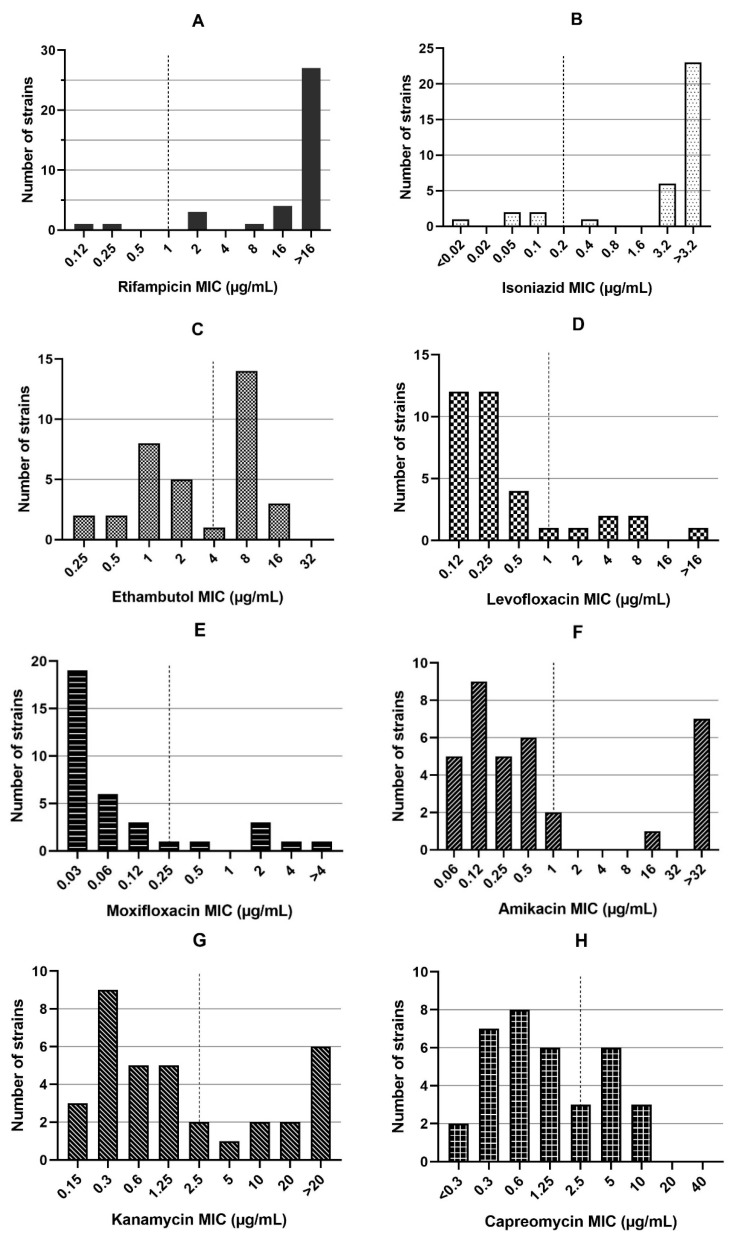

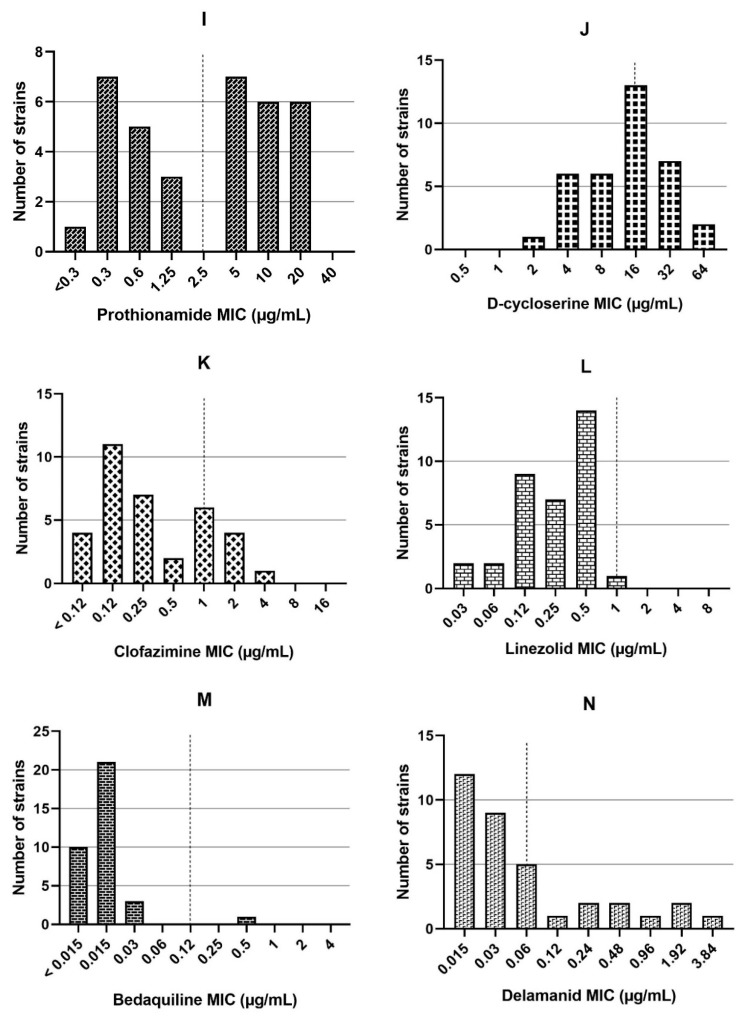
The minimum inhibitory concentrations (MICs) for rifampicin (**A**), isoniazid (**B**), and ethambutol (**C**), levofloxacin (**D**), moxifloxacin (**E**), amikacin (**F**), kanamycin (**G**), capreomycin (**H**), prothionamide (**I**), D-cycloserine (**J**), clofazimine (**K**), linezolid (**L**), bedaquiline (**M**), and delamanid (**N**) in 35 rifampicin-resistant *Mycobacterium tuberculosis* strains. The dash line indicates critical concentrations.

Among the 35 RR-MTB strains, 30 were also resistant to INH. Therefore, they were classified as MDR-TB (85.7%). Resistance to EMB was detected in 17/35 (48.6%) strains. The MIC values of the three first-line and 11 second-line drugs tested are represented in Figure 1. Fluoroquinolone (FLQ) resistance was detected in 6/35 (17.1%) strains, with resistance to both LFX and MFX. Additionally, 8/35 (22.9%), 11/35 (31.4%), and 9/35 (25.7%) strains were found to be resistant to AMK, KAN, and CAP, respectively. Moreover, 19/35 (54.3%) strains were PTO-resistant and 5/35 (14.3%) were CFZ-resistant. In addition, 9/35 (25.7%) strains showed MICs in the resistance range (compared to the *M. tuberculosis* strain H37Rv with MIC = 16 µg/mL) for DCS. However, resistance to LZD was not detected in any strains (0/35, 0%). One (1/35, 2.9%) strain was resistant to BDQ and 9/35 (25.7%) showed phenotypic resistance to DLM.

Overall, 10 pre-extensively drug-resistant (pre-XDR) (MDR-TB also resistant to any FLQ or one of the second-line injectable drugs (SLIDs)) strains and three XDR-TB were found whose resistance pattern is displayed in Table 1. All the pre-XDR/XDR strains were susceptible to LZD, BDQ, and DLM, except two pre-XDR strains (KAN mono-resistant), which were resistant to DLM.

### 3.3. Resistance-Conferring Mutations Using WGS

#### 3.3.1. Genotypic Resistance to the First-Line Drugs

All the RR-MTB strains showed resistance-determinant mutations in *rpoB* gene, except for one (MIC = 2 µg/mL), which was genetically wild type for *rpoB* (Table 2). The most prevalent mutation was Ser531Leu), which was found in 22 (65.7%) strains. Other mutations within the hot spot region of *rpoB* gene were as follows: His526Tyr (four strains), His526Leu (two strains), His526Asp (one strain), Leu533Pro (two strains), Gln513Leu (one strain), Asp516Val (one strain), and Ile572Phe (one strain). Some strains had other substitutions along with well-known *rpoB* mutations, showing mixed wild type/mutant population having several mutations with different frequencies (Table 2). Overall, mutation screening in *rpoB* gene was able to detect 34/35 (97.1%) of rifampicin-resistant strains. Among the INH-resistant strains, 29/30 (96.6%) had a mutation in one of the genes tested, including *katG*, *inhA*, *ahpC*, *fabG1*, *furA*, and their upstream regions. The *katG* Ser315Thr and C-15T at *fabG1*-*inhA* regulatory region were dominant mutations found in 18 (60%) and 10 (33.3%) strains, respectively. An INH-resistant strain (MIC = 3.2 µg/mL) harbored *fabG1* Leu203Leu (57.5% allele frequency) and C-15T at regulatory region of *fabG1*-*inhA* replacement with 50.9% allele frequency in the resistant population. All other mutations associated with isoniazid resistance are presented in Table 2. Moreover, 17/17 (100%) ethambutol-resistant strains showed a mutation in *embA* and *embB* genes. The most frequent mutation was *embB* Met306Val (11 strains). However, three out of 18 EMB-susceptible strains showed *embAB* mutations (Table 2).

#### 3.3.2. Genotypic Resistance to Fluoroquinolones

Five (83.3%) out of six LFX/MFX-resistant strains had mutations in the *gyrA* gene, including Gly88Cys (two strains), Asp94Gly (two strains), and Ser91Pro (one strain). However, an LFX/MFX susceptible strain (MICs on critical concentration for both drugs) showed a *gyrA* Asp94Ala mutation. There was no mutation in quinolone-resistant determining region (QRDR) of the *gyrB* gene (Table 3).

#### 3.3.3. Genotypic Resistance to Second-Line Injectable Drugs

All mutations found in *rrs*, *eis*, and *tlyA* genes associated SLIDs resistance are shown in Table 4. The A1401G (eight strains) mutation was the dominant mutation in *rrs* gene, followed by G1484T (one strain). In addition to the *rrs* gene, four different mutations were found in *eis* promoter (C-10T, G-12A, G-14A, and G-15C), indicating low-level resistance to KAN. However, strain with a G-14A mutation, was phenotypically susceptible to KAN. In the case of CAP, a C insertion at position 210 of *tlyA* gene was found in a CAP-resistant strain along with eight strains having *rrs* mutations. Hence, 8/8 (100%), 11/11 (100%), and 9/9 (100%) strains showed resistant genotype for AMK, KAN, and CAP, respectively. Nevertheless, Gly196Glu mutation of *tlyA* gene was observed in a CAP-susceptible strain.

#### 3.3.4. Genotypic Resistance to Other Second-Line Drugs (WHO Group C)

The mutations found in the genes associated with resistance to PTO, DCS, CFZ, and LZD are detailed in Table 5. Among the 19 PTO-resistant strains, mutations in *ethA*, *ethA*-ups, *inhA*, and *fabG1*-ups were detected in all (19/19, 100%) strains, of which 10 showed a *fabG1*-ups C-15T substitution. Furthermore, two susceptible strains had a *ethA* Gln254Pro mutation. All the nine DCS-resistant strains were wild type, except for one strain (1/9, 11.1%) that showed *cycA* Pro188Ala mutation. Additionally, 10 DCS-susceptible strains were found to have different mutations in targeted genes, of which seven showed mixed population. Moreover, six strains had MICs = 16 µg/mL, identical to the H37Rv strain (Table 5). All the genes involved in resistance to CFZ were wild type in CFZ-resistant strains, except *Rv1979c*, which showed adenine deletion at position 1014 (1/5, 20%). However, the MICs of three strains with various substitutions were on breakpoint (1 µg/mL). Two strains with *rrl* mutations (C1537T and C1331T) and two with a *rplC* Ala72Thr mutation were susceptible to LZD (Table 5).

#### 3.3.5. Genotypic Resistance to Add-On Agents

The only mutation detected in BDQ resistance-related genes was *Rv0678* Gly87Arg, which was found in a BDQ-susceptible strain. The *atpE*, *atpE*-ups (upstream), and *Rv0678*-ups genes did not harbor any mutations in any strain. A BDQ-resistant strain (MICs = 0.5 µg/mL) was found as wild type in studied genes (0/1, 0%). Three different mutations (G-deletion at position 91 of *ddn*, *fbiA* Arg321Ser (two strains) and *fbiC* Trp678Gly) were found in 4/9 (44.4%) of DLM-resistant strains. However, *fbiC* Trp678Gly mutation was also identified in a DLM-susceptible strain.

### 3.4. Sensitivity, Specificity, and Accuracy of WGS

WGS showed the highest sensitivity in detecting resistance to EMB, PTO, and SLIDs (100%). Additionally, the lowest sensitivity was found in the case of BDQ, DCS, and CFZ by 0.0%, 11.1%, and 20.0%, respectively. Table 6 summarizes the sensitivity, specificity, and overall accuracy of WGS for all tested drugs, compared with pDST.

### 3.5. WGS-Based MTB Genotyping

**Figure 2:**
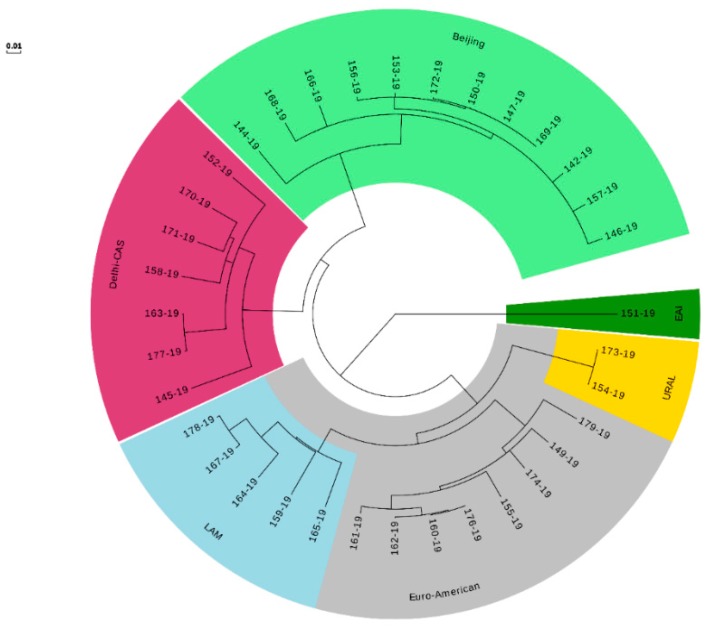
Maximum likelihood tree built from 544 single nucleotide polymorphism (SNP) positions of the 35 rifampicin-resistant *Mycobacterium tuberculosis* isolates mapped to the genome of H37Rv (NC_000962.3) shown as a circular phylogram.

WGS confirmed the heterogeneous TB epidemiology among our isolates with the four major MTB complex lineages. Lineage 1; East-African Indian (includes EAI and EAI Manila), Lineage 2; East-Asian (includes Beijing), Lineage 3; Indo-Oceanic (includes Delhi-CAS), and Lineage 4; Euro-American (includes Cameroon, Haarlem, H37Rv-like, LAM, mainly T, S-type, Ural and X-type). Most represented sublineages were Beijing, Euro-American (sublineage 4.5), and Delhi-CAS (Figure 2). Cluster analysis using the threshold of five SNPs as maximum distance between group members identified three distinct clusters (A, B, and C). Four pre-XDR strains (No. 147-19, 150-19, 169-19, 172-19) from three provinces from west to the capital of Iran classified in cluster A (Beijing). Cluster B (Ural) includes two MDR-MTB strains (154-19, 173-19) recovered from two patients in the capital. Strains in cluster A and B were isolated during several years and might distributed due to referring drug-resistant patients from other cities to the capital for treatment and following. Two pre-XDR/XDR strains (160-19, 176-19) isolated from members of a family during a year were placed in cluster C (Figure 2). The genotype of five remaining pre-XDR strains were Beijing (two strains from the west), Euro-American (one strain from the capital and one strain from the Afghani patient at south), and LAM (1 strain, Capital). An XDR-TB patient was from the north (Beijing) and another XDR strain isolated from the Iraqi patient at south-west of Iran (LAM). Among the RR-MTB strains, three Euro-American, one EAI, and one Delhi-CAS lineages/sublineages were detected in patients from the capital.

## 4. Discussion

To determine the MIC distributions of first-/second-line anti-TB drugs among RR/MDR-MTB strains, phenotypic DST was performed using the broth microdilution method. Different methods are available to determine the MICs and based on the results and some other factors including, cost, labor requirements, published MIC data, and experiences from other pathogens, Middlebrook 7H9 was also proposed as the EUCAST reference method for MTBC [34]. Additionally, WGS analysis was performed on 35 collected RR-MTB strains to screen genetic mutations in drug-resistance-related genes.

In the case of rifampicin, accuracy of the WGS to detect rifampicin-resistant strains was 97.1%, and regardless of strains with mixed mutations, all the mutations were located at 81-bp rifampicin-resistant determinant region (RRDR) of the *rpoB* gene, except for one (Ile572Phe). Thus, screening only the 81-bp region could detect 94.3% of rifampicin-resistant strains that some resistant strains with no mutation in this region have previously shown [35,36]. In addition, there were no strains having mixed mutations within the RRDR. In accordance with the previous studies from different geographical regions [37,38,39], codon 531 of the *rpoB* gene was the most abundant mutation in this study. Moreover, 85.7% of the RR-MTB strains were found to be resistant to INH (MDR-TB), and testing only the *katG* gene could detect 83.3% of the strains. However, 83.3% of INH-resistant strains had *katG* Ser315Thr and *fabG1*-*inhA* C-15T mutations which could have been increased to 96.6% if the sequence analysis included the entire *katG* gene. These data were supported by other studies [40,41,42,43,44]. The *ahpC*, and *furA* structural genes were wild type in strains examined in this study and had a less role in resistance to INH, as limited data and mutations with lower incidence of these genes have been reported in previous studies [42,43,45,46]. Ethambutol resistance was mostly linked to the mutations within the *embB* gene (codon 306 as the dominant position), but not *embA*. Khosravi et al. reported *embA* mutation from EMB-susceptible strains [47]. Additionally, Sun et al revealed that 82.6% of EMB-resistant isolates from China had at least a mutation in the *embABC* locus, but contrary to this study, they were located mostly in the *embA* and *embB* upstream region [48].

Sequencing results of *gyrA* gene covered five FLQ resistance (three XDR and two pre-XDR strains) with the accuracy of 94.3%, and the QRDR of the *gyrB* gene was wild type in all strains. The *gyrA* Asp94Ala, detected in an LFX/MFX susceptible strain with MICs equal to 1 and 0.25 µg/mL. This mutation may lead to only ciprofloxacin resistance or is associated with lower MICs to FLQ than other substitutions within this codon, as was reported in the literature [26,49]. In addition, *gyrA* Gly88Cys was more associated with higher MICs for both LFX and MFX than other detected mutations. A strain with low-level resistance to LFX (MIC = 2 µg/mL) and MFX (MIC = 0.5 µg/mL) did not harbor any mutation in the related genes, suggesting additional resistance mechanisms, such as efflux pumps [50].

Testing for only the *rrs* gene could detect all eight AMK/KAN/CAP-R phenotypes, including three XDR and five SLIDs resistant (pre-XDR) strains and resistance to KAN, and CAP was correctly diagnosed in all strains using *rrs*, *eis*-ups, and *tlyA* genes. In concordance with previous studies, two KAN mono-resistant strains (low-level resistance with MIC = 10 µg/mL) were found to harbor C-10T and G-15C substitutions at the *eis* promoter [51,52,53]. However, Pholwat et al. indicated that the G-15C mutation does not confer KAN resistance in *M. tuberculosis* [54]. A KAN/CAP-resistant strain, with MIC = 5 µg/mL for both drugs, possesses *eis* G-12A and *tlyA* C210 insertion. The only strain with a G-14A mutation at the *eis* promoter was phenotypically susceptible to KAN (MIC = 0.3 µg/mL). This mutation was investigated as KAN-resistance conferring mutation in literature [51,52]. Furthermore, *tlyA* Gly196Glu was found in a CAP-susceptible strain that may not determine CAP resistance, while Gly196Glu was previously reported in resistant isolates [30].

Approximately 54% of rifampicin-resistant strains were identified as PTO-resistant in the present study. Additionally, the sensitivity and specificity of PTO resistance detection were 100% and 87.5%, respectively, using WGS. Cross resistance among PTO and INH was noted in 94.7% of PTO-resistant strains. Similar to the previous studies, in this study, more than half of the total mutations in PTO-resistant phenotypes were found in the *inhA*/*fabG1* regulatory regions [55,56]. The *ethA* Gln254Pro mutation was found in two susceptible phenotypes, while the later mutation was also identified in two resistant strains (MIC = 5, 10 µg/mL). Nevertheless, the Gln254Pro substitution was previously reported from ethionamide-resistant *M. tuberculosis* strains [57]. Surprisingly, DCS resistance-related genes targeted in this study showed a single *cycA* Pro188Ala mutation in one of nine strains recognized as DCS-resistant (the low sensitivity by 11.1%). Despite this, 10 out of 26 DCS-susceptible strains harbored different mutations and most of them, with MICs equal to or less than what was observed in the H37Rv, showed mixed wild type/mutant population. Results of this study emphasized the controversial role of *alr*, *ald*, *ddlA*, and *cycA* genes in molecular detection of DCS resistance. Previous attempts indicated complex mechanisms in resistance to this drug [58,59]. Only the *alr* D344N substitution, identified by Chen et al., and novel mutations in genes other than known DCS resistance-associated genes have been reported [59]. WGS, with low sensitivity (20%), traced only A1014 deletion of *Rv1979c* among five CFZ-resistant strains. The reason for this low sensitivity is that currently, BDQ and CFZ do not yet have well-defined mechanism of resistance or well-documented genomic targets [60]. However, three strains, with MICs on breakpoint (MIC = 1 µg/mL), had various mutations in three candidate genes, including *Rv0678*, *Rv1979c*, and *pepQ*. These results were inconsistent with those of the previous studies. Zhang et al. showed that 97% of the CFZ-resistant mutants had a mutation in the *Rv0678* gene, encoding the MmpR repressor [61]. Additionally, mutations in these three genes and their upstream region were reported in the CFZ-resistant strains [32,61].

In the present study, LZD and BDQ were the most effective drugs against MDR/pre-XDR and XDR strains of *M. tuberculosis*. However, unlike the present data, 10% LZD resistance in MDR/XDR isolates was previously reported from Iran [62]. Different mutations in *rrl* and *rplC* genes had a role in LZD resistance [32], while the four mutant strains in the current study, with C1537T and C1331T substitutions in the *rrl* gene and *rplC* Ala72Thr mutation (two strains), were LZD-susceptible. In addition, Yang et al. reported several *rrl* mutations in LZD-susceptible strains with MIC below 0.5 mg/L [63]. There was no cross resistance between CFZ and BDQ in the tested strains in this study, and the only *Rv0678* Gly87Arg mutant was also susceptible to BDQ (MIC = 0.01 µg/mL). The *atpE* gene was wild type in all strains. Meanwhile, the strain with MIC greater than the epidemiological cut-off (0.12 µg/mL, reported by the European Committee on Antimicrobial Susceptibility Testing- EUCAST) were genetically wild type. Thus, the role of the *Rv0678* and *atpE* in resistance is controversial, as mutations of these genes in susceptible and resistant strains have been reported. In other words, both susceptible and BDQ-resistant strains may carry mutations in these genes and the presence of mutations does not necessarily indicate resistance [32,63,64].

Among the rifampicin-resistant strains, 25.7% were resistant to DLM. In other words, three rifampicin-resistant, four MDR, and two pre-XDR strains were resistant to DLM and all three XDR strains were susceptible to DLM. In addition, DLM-resistant strains were belonged to different lineages/sublineages including Beijing (three strains), Euro-American (three strains), Delhi-Cas (three strains). Additionally, six of them were isolated from same city and the remaining three strains obtained from two different cities. As the first investigation, the high rate of DLM resistance was amazingly considerable, while this new compound has not yet been used as a treatment choice of TB patients in Iran. Moreover, the sensitivity of WGS in DLM resistance detection was almost low (44.4%). The *fbiA* Arg321Ser was the common mutation found in two (22.2%) DLM-resistant strains. However, *fbiC* Trp678Gly mutant was found in an DLM-susceptible phenotype. Molecular basis of resistance to DLM is still poorly understood, and the present results around the DLM MICs and genetic background of its resistance-associated genes are in accordance with previous studies [33,63].

## 5. Conclusions

The highest resistance rate among RR/MDR strains of *M. tuberculosis* was found in PTO. However, LZD and BDQ were found as the most effective drugs against all kinds of drug-resistant phenotypes (RR/MDR/pre-XDR/XDR). It was surprising to find more than 25% DLM resistance among the drug-resistant phenotypes without exposure to this agent. WGS showed the lowest sensitivity for detecting resistance to BDQ (0.0%), DCS (11.1%), and CFZ (20.0%), followed by DLM (44.4%) which is related to the current poor knowledge of genetic variants related to the resistant phenotype. Additionally, the highest sensitivity among the second-line drugs belonged to PTO and SLIDs (100.0%). Finally, mutations in drug resistance-related genes were mostly linked to the MICs results, especially for key anti-TB drugs; hence, the RR/MDR/pre-XDR/XDR strains can be identified with high confidence using WGS. However, if fully susceptible strains were present in this study, a better interpretation of the mutations could be achieved. In addition, limited data around the molecular background and resistance mechanisms of some drugs, especially new compounds, was another restriction.

## Figures and Tables

**Table 1:** Resistance pattern of pre-extensively drug-resistant (pre-XDR) and extensively drug-resistant (XDR) strains.

	No. of Strains	LFX	MFX	AMK	KAN	CAP
**Pre-XDR**	3	R	R	S	S	S
	4	S	S	R	R	R
	2	S	S	S	R	S
	1	S	S	S	R	R
**XDR**	3	R	R	R	R	R

LFX: levofloxacin, MFX: moxifloxacin, AMK: amikacin, CAP: capreomycin, pre-XDR: pre-extensively drug-resistant, XDR: extensively drug-resistant, R: resistant, S: susceptible.

**Table 2:** Resistance-conferring mutations for the first-line anti-tuberculosis (anti-TB) drugs in 35 rifampicin-resistant *Mycobacterium tuberculosis* strains [3,26,27,28,29].

Drug	Resistance Phenotype (No. of Strains)	Resistance Genotype			MIC (µg/mL)	Total
		Gene	Mutations	No. of Strains		
**RIF**	Resistant (35)	*rpoB*	Ser531Leu	19	>16	34/35
			Ser531Leu/Arg862Trp (freq: 19.9%)	1	>16	
			Ser531Leu/Val643Ala (freq: 38.5%)	1	>16	
			Ser531leu/Pro564Ser (freq: 12.6%)	1	>16	
			His526Tyr	2	>16	
				2	16	
			His526Leu	2	16	
			His526Asp/Ser512Thr (freq: 5.6%)	1	>16	
			Leu533Pro	2	4	
			Gln513Leu	1	>16	
			Asp516Val	1	>16	
			Ile572Phe	1	8	
			WT	1	2	
**INH**	Resistant (30)	*katG*	Ser315Thr	11	>3.2	29/30
				2	3.2	
			1353 to 1476-del	1	>3.2	
			Ser315Thr/Leu216Pro	1	3.2	
		*katG*/*ahpC*-ups	Asn138His/T-76A	1	>3.2	
		*katG*/*ahpC*-ups	Ser315Thr/T-77G	1	>3.2	
		*katG/ahpC*-ups/*fabG1*-ups/*inhA*	Trp149Arg/G-51A/ −27 to −38 del/Ser94Ala	1	>3.2	
		*katG*/*ahpC*-ups/*inhA*	Trp149Arg/G-51A/Ser94Ala	1	>3.2	
		*katG/fabG1*-*inhA*	Met624Ile/C-15T	1	>3.2	
		*katG*/*fabG1*-*inhA*	Ser315Thr/C-15T	3	>3.2	
		*katG*/*fabG1*-*inhA*	Leu587Pro/C-15T	1	>3.2	
		*katG*/*fabG1*-*inhA*	Trp191Arg, Ala706Thr/C-15T	1	>3.2	
		*inhA/fabG1-inhA*	Ala239Val/C-15T	1	>3.2	
		*fabG1/fabG1-inhA*	Leu203leu (freq: 57.5%)/C-15T (freq: 50.9%)	1	3.2	
		*fabG1*-*inhA*	C-15T	1	3.2	
				1	0.4	
		*katG*, *inhA*, *ahpC*, *fabG1*, *furA*	WT	1	3.2	
**EMB**	Resistant (17)	*embB*	Met306Val	10	8	17/17
				1	16	
			Gly406Ser	1	16	
			Gln497Arg	1	8	
			Met306Ile/Gly406Asp	1	16	
		*embB*/*embA*-ups	Met306Ile/C-16A	1	8	
		*embB*/*embA*-ups	Met306Ile+Trp349Arg/C-16A	1	8	
		*embB*/*embA*-ups	Gln497Arg/C-12T	1	8	
	Susceptible (18)	*embA*-ups	C-16T	1	2	3/18
		*embB*	His1002Arg	1	1	
			Asp328Gly	1	4	

MIC: minimum inhibitory concentration, RIF: rifampicin, INH: isoniazid, EMB: ethambutol, ups: upstream, del: deletion, WT: wild type, freq: frequency, A; adenine, T; thymine, G; guanine, C; cytosine.

**Table 3:** Fluoroquinolone resistance determinant mutations in 35 rifampicin-resistant *Mycobacterium tuberculosis* strains [26,27].

Drug	Resistance Phenotype (No. of Strains)	Resistance Genotype			MIC (µg/mL)	Total
		Gene	Mutations	No. of Strains		
**LFX**	Resistant (6)	*gyrA*	Gly88Cys	1	>16	5/6
				1	8	
			Asp94Gly	2	4	
			Ser91Pro	1	8	
		*gyrA*, *gyrB*	WT	1	2	
	Susceptible (29)	*gyrA*	Asp94Ala	1	1	1/29
**MFX**	Resistant (6)	*gyrA*	Gly88Cys	1	>4	5/6
				1	4	
			Asp94Gly	2	2	
			Ser91Pro	1	2	
		*gyrA*, *gyrB*	WT	1	0.5	
	Susceptible (29)	*gyrA*	Asp94Ala	1	0.25	1/29

MIC: minimum inhibitory concentration, LFX: levofloxacin, MFX: moxifloxacin, WT: wild type.

**Table 4:** Mutations found in genes associated with resistance for second-line injectable agents in 35 rifampicin-resistant *Mycobacterium tuberculosis* strains [3,26,27,30].

Drug	Resistance Genotype (No. of Strains)	Resistance Genotype			MIC (µg/mL)	Total
		Gene	Mutations	No. of Strains		
**AMK**	Resistant (8)	*rrs*	A1401G	6	>32	8/8
				1	16	
			G1484T	1	>32	
**KAN**	Resistant (11)	*rrs*	A1401G	5	>20	11/11
				2	20	
			G1484T	1	>20	
		*eis*-ups	G-10A	1	10	
			C-12T	1	5	
			C-15G	1	10	
	Susceptible (24)	*eis/eis*-ups	G334 ins/C-14T	1	0.3	1/24
**CAP**	Resistant (9)	*rrs*	A1401G	5	5	9/9
				2	10	
			G1484T	1	10	
		*tlyA*	C210 ins	1	5	
	Susceptible (26)	*tlyA*	Gly196Glu	1	2.5	1/26

MIC: minimum inhibitory concentration, AMK: amikacin, KAN: kanamycin, CAP: capreomycin, ups: upstream, ins: insertion, A; adenine, T; thymine, G; guanine, C; cytosine.

**Table 5:** Mutations found in resistance-related genes for the World Health Organization (WHO) group C second-line drugs in in 35 rifampicin-resistant *Mycobacterium tuberculosis* strains [3,26,28,31,32,33].

Drug	Resistance Phenotype (No. of Strains)	Resistance Genotype			MIC (µg/mL)	Total
		Gene	Mutations	No. of Strains		
**PTO**	Resistant (19)	*ethA*	Thr314Ile	1	5	19/19
			Ala341Glu	1	5	
			Trp21 del (freq: 10.32%)	1	5	
			T1361 del	1	10	
			Gln254Pro	1	10	
				1	5	
		*ethA*/*fabG1*-ups/*inhA*	Trp116 del (freq: 59.63%)/−27 to −38 del/Ser94Ala (freq: 20.69%)	1	20	
		*ethA*-ups	T-11G	1	10	
		*fabG1*-*inhA*	C-15T	2	20	
				3	10	
				3	5	
		*inhA*/*fabG1*-*inhA/ndh*	Ala239Val/C-15T/399A ins	1	20	
		*fabG1/fabG1-inhA*	Leu203Leu (freq: 57.5%)/C-15T (freq: 50.9%)	1	20	
		*inhA*	Ser94Ala	1	20	
	Susceptible (16)	*ethA*	Gln254Pro	2	0.6	2/16
**DCS**	Resistant (9)	*cycA*	Pro188Ala	1	32	1/9
		*ald, ddlA, alr, cycA*	WT	6	32	
			WT	2	64	
	Susceptible (26)	*ald*	GC432 ins	2	16	10/26
			Cyc333Trp (freq: 10.9%)	1	16	
			Ala340Ser (freq: 5.0%)	1	16	
		*ddlA*	2A ins (freq: 7.3%)	1	4	
			Arg264Gly (freq: 6.5%)/Val260Gly (freq: 6.0%)	1	16	
			Gln293Glu (freq: 5.0%)	1	8	
			Thr322Ser (freq: 5.1%)	1	2	
		*alr*	Leu113Arg	1	16	
		*cycA*	Leu21His (freq: 5.3%)	1	8	
**CFZ**	Resistant (5)	*Rv1979c*	1014A del	1	2	1/5
		*Rv0678, Rv1979c, pepQ*	WT	3	2	
			WT	1	4	
	Susceptible (30)	*Rv0678*	Gly87Arg	1	1	3/30
		*Rv1979c*	Asn297Ser	1	1	
		*pepQ*	Ala87Gly (freq: 12.79%)	1	1	
**LZD**	Susceptible (35)	*rrl*	C1537T	1	0.25	4/35
			C1331T	1	0.12	
		*rplC*	Ala72Thr	2	0.5	

WHO: World Health Organization, MIC: minimum inhibitory concentration, PTO: prothionamide, DCS: D-cycloserine, CFZ: clofazimine, LZD: linezolid, ups: upstream, del: deletion, WT: wild type, ins: insertion, freq: frequency, A; adenine, T; thymine, G; guanine, C; cytosine.

**Table 6:** Sensitivity, specificity, and overall accuracy of whole genome sequencing compared to the phenotypic drug susceptibility testing.

Drug	Sensitivity (%)	Specificity (%)	Accuracy (%)
**Rifampicin**	97.1	100.0	97.3
**Isoniazid**	96.7	100.0	97.1
**Ethambutol**	100.0	83.3	91.4
**Levofloxacin**	83.3	96.5	94.3
**Moxifloxacin**	83.3	96.5	94.3
**Amikacin**	100.0	100.0	100.0
**Kanamycin**	100.0	95.8	97.1
**Capreomycin**	100.0	96.2	97.1
**Prothinamide**	100.0	87.5	94.3
**D-cycloserine**	11.1	61.5	48.6
**Clofazimine**	20.0	90.0	80.0
**Linezolid**	-	88.6	88.6
**Bedaquiline**	0.0	97.1	94.3
**Delamanid**	44.4	96.2	82.9
